# Bisphosphonate Use May Reduce the Risk of Urolithiasis in Astronauts on Long‐Term Spaceflights

**DOI:** 10.1002/jbm4.10550

**Published:** 2021-09-22

**Authors:** Atsushi Okada, Toshio Matsumoto, Hiroshi Ohshima, Tatsuya Isomura, Tadashi Koga, Takahiro Yasui, Kenjiro Kohri, Adrian LeBlanc, Elisabeth Spector, Jeffrey Jones, Linda Shackelford, Jean Sibonga

**Affiliations:** ^1^ Department of Nephro‐urology Nagoya City University Graduate School of Medical Sciences Nagoya Japan; ^2^ Fujii Memorial Institute of Medical Sciences Tokushima University Tokushima Japan; ^3^ Japan Aerospace Exploration Agency Tsukuba Japan; ^4^ Institute of Medical Science Tokyo Medical University Tokyo Japan; ^5^ Department of Pharmacology St. Marianna University School of Medicine Kawasaki Japan; ^6^ Baylor College of Medicine‐ Center for Space Medicine Houston TX USA; ^7^ KBR Wyle Houston TX USA; ^8^ NASA Johnson Space Center Houston TX USA

**Keywords:** BONE MODELING AND REMODELING, BIOCHEMICAL MARKERS OF BONE TURNOVER, DISEASES AND DISORDERS OF/RELATED TO BONE, OSTEOPOROSIS, THERAPEUTICS, ANTIRESORPTIVES, KIDNEY STONE, RENAL STONE, URINARY CALCULUS, NEPHROLITHIASIS, UROLITHIASIS, SPACEFLIGHT/ASTRONAUTS

## Abstract

Long‐duration spaceflight is associated with an increased risk of urolithiasis, and the pain caused by urinary calculi could result in loss of human performance and mission objectives. The present study investigated the risk of urolithiasis in astronauts during 6 months on the International Space Station, and evaluated whether the suppression of bone resorption by the bisphosphonate, alendronate (ALN), can reduce the risk. A total of 17 astronauts were included into the analysis: exercise using the advanced resistive exercise device (ARED) plus weekly oral 70 mg alendronate (ARED+ALN group, *n* = 7) was compared to resistive exercise alone (ARED group, *n* = 10). Urine volume decreased in both groups during spaceflight but recovered after return. The ARED group showed increased urinary calcium excretion from the 15th to 30th day of spaceflight, whereas urinary calcium was slightly decreased in the ARED+ALN group. Urinary N‐terminal telopeptide (NTX) and helical peptide (HP) of type I collagen, as bone resorption markers, were elevated in the ARED group during and until 0 days after spaceflight, while there was no elevation in these parameters in the ARED+ALN group. Urinary oxalate and uric acid excretion tended to be higher in the ARED group than in the ARED+ALN group during spaceflight. These results demonstrate that astronauts on long‐duration spaceflights may be at high risk for the formation of urinary calcium oxalate and calcium phosphate stones through increased urinary excretion of oxalate and uric acid, from degraded type I collagen, as well as of calcium from enhanced bone resorption. Our findings suggest that increased bone resorption during spaceflight, as a risk factor for urinary calculus formation, could be effectively prevented by an inhibitor of bone resorption. © 2021 The Authors. *JBMR Plus* published by Wiley Periodicals LLC on behalf of American Society for Bone and Mineral Research.

## Introduction

The prevalence of urolithiasis appears to be increasing worldwide. In the United States alone, the prevalence in men and women is 10.6% and 7.1%, respectively.^(^ ^1^ ^)^ The elevated excretion of bone resorption biomarkers consistently documented in astronauts during spaceflight^(^ ^2^, ^3^, ^4^ ^)^ suggests that astronauts are at risk for urinary calculi during spaceflight. In addition to the potential impact to crew health, the formation of a stone during spaceflight could negatively influence the performance of mission objectives and overall mission success.^(^ ^5^ ^)^

Cockett, Beehler, and Roberts^(^ ^6^ ^)^ in 1962 were the first to publish a description of weightlessness as a potential hazard to astronauts, due to increased calcium excretion resulting in the formation of urinary calculi. In a microgravity environment the mechanical loading of bones and muscles is removed. There is a resulting imbalance in bone remodeling, with bone resorption exceeding bone formation, as an adaptive skeletal response to reduced biomechanical stress. As a result, femoral bone mineral density (BMD) decreases by about 1.5% per a month in space, which is about 10 times the rate of bone loss in postmenopausal women.^(^ ^7^ ^)^ As mentioned, enhanced bone resorption can be associated with increased urinary calcium.

To counter this increased urinary stone risk during spaceflight, potassium citrate administration to crewmembers of the Russian Mir space station and the International Space Station (ISS) was shown to reduce urinary calcium excretion and to reduce the risk of calcium oxalate supersaturation.^(^ ^8^ ^)^ This is in agreement with a recent retrospective clinical study that potassium‐sodium citrate prevented the development of renal microcalculi into symptomatic stones in calcium stone‐forming patients.^(^ ^9^ ^)^ It has been reported that heavy resistive exercise on the ISS Advanced Resistive Exercise Device (ARED), along with adequate vitamin D levels and energy intake, is sufficient to maintain bone mass at the preflight level. However, the increase in urinary calcium during spaceflight was not prevented in those astronauts, and use of ARED was not sufficient to block the increase in bone resorption markers.^(^ ^3^, ^4^ ^)^ However, those studies did not include the data on the effect of alendronate (ALN) on urinary stone risks.^(^ ^2^, ^3^, ^4^ ^)^

Our previous studies showed a decrease in BMD and an increase in urinary calcium excretion and renal stone risk by long‐term bed rest for 90 days.^(^ ^10^, ^11^ ^)^ In those studies, the increase in urinary calcium excretion as well as the reduction in BMD were markedly ameliorated by a single injection of a bisphosphonate, pamidronate, before bed rest. Based on those results, and those of LeBlanc and colleagues,^(^ ^12^ ^)^ a spaceflight study was performed to investigate the effect of bisphosphonate in reducing the risks of bone mineral loss and urinary stone formation during long‐duration spaceflight as a collaborative effort between the National Aeronautic Space Administration (NASA) and the Japan Aerospace Exploration Agency (JAXA). It is suggested that the combination of ARED plus bisphosphonate showed an additive effect to exercise alone for mitigating declines in BMD during long‐duration space missions.^(^ ^13^, ^14^ ^)^ However, although all the astronauts taking ALN were under ARED exercise, because ARED exercise apparatus was just introduced at the time the study was performed, serum and urinary data of most of control astronauts were obtained from astronauts under pre‐ARED (interim Resistive Exercise Device, iRED) exercise. Therefore, the effect of ALN+ARED in comparison with ARED control on urinary stone risk factors has not been examined previously.

The present study was undertaken to assess the urolithiasis‐related variables of astronauts on the ISS for 6 months, and to examine whether alendronate (ALN) administration along with ARED exercise can also reduce the risk of renal stone formation in astronauts.

## Subjects and Methods

### Subjects

The study was reviewed and approved by the NASA Johnson Space Center Institutional Review Board (IRB) and JAXA IRB for Human Research, and all protocols complied with the World Medical Association Declaration of Helsinki—Ethical Principles for Medical Research Involving Human Subjects. Written informed consent was obtained and all astronauts provided consent to participate this study.

The study included 17 astronauts, and they were divided into the following two groups: (i) exercise plus medication group (ARED+ALN group; *n* = 7), astronauts received oral alendronate (70 mg/week; Fosamax® starting 3 weeks before space flight and continuing throughout the flight) and exercised according to a prescribed protocol; and (ii) exercise alone group (ARED group; *n* = 10), astronauts only exercised according to a prescribed protocol. Details regarding medication have been described.^(^ ^13^, ^14^ ^)^ The subject characteristics are described in Table 1. The mission length varied between 4.5 and 6.2 months (mean mission length of 5.5 months). Astronauts in the ARED+ALN group were instructed to take alendronate after an overnight fast with 236 mL of water and not to eat or drink for 30 minutes after medication ingestion. The medication was packed in foil push‐out cards labeled for each astronaut. A reminder was sent to the astronaut on the designated day of ingestion. The timing of first food and drink intake after medication ingestion was recorded on a comment card, and the astronauts were instructed to report any anomalies with regard to the date and time of medication ingestion. No anomalies were noted, and there was >98% compliance with regard to the dosing schedule throughout the study.

**Table 1:** Subject Characteristics and Renal Calcification Data

Characteristic	ARED group (*n* = 10)	ARED+ALN group (*n* = 7)	*p* ^a^
Age (years), mean ± SD	45.0 ± 6.5	49.4 ± 3.7	0.0943
Gender (male/female), *n*	9/1	5/2	0.5368
Renal calcification (yes/no), *n*	1/9	0/7	1.0000

ARED = exercise; ARED+ALN = exercise plus alendronate; SD = standard deviation.

^a^ Age was compared by *t* test whereas gender and renal calcification were compared by Fisher's exact test.

Strength, conditioning, and rehabilitation specialists are assigned to each astronaut, and they cooperate with astronauts to create plans for individual spaceflight exercise. Details regarding exercise have been described.^(^ ^13^, ^14^ ^)^ The exercise plan during spaceflight is designed to maintain or exceed the astronaut's preflight fitness level. A total of 2.5 hours per day is allotted for exercise 6 days a week where the 2.5‐hour allocation includes time for device setup and personal hygiene. The exercise regimen performed by the astronaut during spaceflight, as prescribed by the astronaut strength and conditioning specialist on the ground, is a combination of both resistive exercise on the ARED and aerobic exercises on the cycle ergometer or with treadmill running.

### Detection of urinary calculi

Abdominal ultrasound was performed using the HDI‐5000 Multipurpose Ultrasound System (ATL Inc., Bothell, WA, USA) or a comparable device before and after space flight to evaluate the abdominal organs, liver, spleen, pancreas, abdominal aorta, inferior vena cava, gallbladder, biliary duct, kidneys, ureters, bladder, Morrison's pouch, and main portal vein.

### Sample collection and biochemical analyses

Twenty‐four‐hour urine samples were collected (i) before spaceflight (PRE), (ii) during spaceflight on the 15th, 30th, 60th, 120th, and 180th flight day (FD) aboard the ISS (denoted as FD15, FD30, FD60, FD120, and FD180, respectively), and after spaceflight on landing day, 30 days, and 1 year after recovery (denoted as R0, R30, and R1yr, respectively). For astronauts who returned from the ISS earlier than FD180, urine samples for FD180 were collected 7 days before return. Preflight urine samples were obtained at approximately 45 days before spaceflight. Urine samples were analyzed for urine volume (UV), pH, urinary creatinine (Cr), N‐terminal telopeptide (NTX), helical peptide (HP), calcium (Ca), phosphorus (P), magnesium (Mg), uric acid (UA), oxalate, and citrate. Additionally, relative supersaturation (RSS) values were calculated using the Finlayson method^(^ ^15^ ^)^ for calcium oxalate, calcium phosphate (apatite and brushite), monosodium urate, struvite, and uric acid to assess the renal stone formation risk of each component. These unit‐free ratios were determined from the solubility product of the various concentrations of the chemical composition and represent the saturation of stone‐forming salts and the concentration of undissociated uric acid. Supersaturation data are expressed relative to values in normal, non‐stone‐forming subjects and indicate the state of urinary supersaturation, which is a fundamental requirement for stone formation. Although a previous work defined a supersaturation ratio >1 as an indication of an increased risk of calcium oxalate, calcium phosphate, monosodium urate, and uric acid stone formation,^(^ ^15^ ^)^ in order to define the risk more solidly, we usually use a supersaturation ratio >2 as an increased risk of those stone formation. Values >75 reflect an increased risk of struvite stones.^(^ ^16^ ^)^

### Statistical analysis

All analyses were performed using SAS Release9.4 (SAS Institute Inc., Cary, NC, USA). Values of *p* <0.05 were considered statistically significant, and all tests were conducted two‐sided.

The data were repeatedly measured over the study time period for each subject. Therefore, a general linear mixed model was applied using the PROC MIXED procedure in SAS. The model included treatment group, time, and their interaction as fixed effects, as well as subject as a random effect, and preflight value as a covariate. Within‐subject change from preflight and between‐groups difference at each time point were assessed by the model, respectively. For comparison purpose, figures were also created to show relationship between change (%) from preflight and time points based on the estimates from the above model, supplementally.

## Results

Subject backgrounds and the results of abdominal ultrasonography are summarized in Table 1. No significant differences were detected between the groups. A renal calcification was observed before spaceflight in one astronaut from the ARED group, which still remained after spaceflight without change in its size (images not shown).

**Fig. 1:**
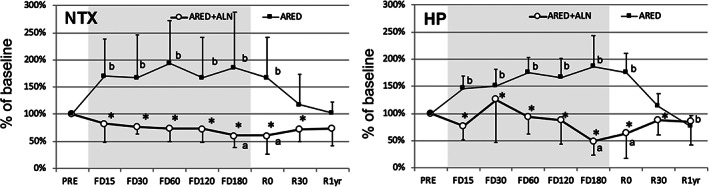
Percent changes from preflight in bone resorption markers. Dots indicate mean and error bars standard deviation of change (%) from preflight calculated using estimates from a linear mixed effects model. ^a^ *p* < 0.05 versus PRE in ARED+ALN, ^b^ *p* < 0.05 versus PRE in ARED, *p < 0.05 between the raw values of ARED+ALN and ARED. ARED = exercise alone; ARED+ALN = exercise plus alendronate; HP = urinary helical peptide; NTX = cross‐linked N‐telopeptide of type I collagen.

Daily urine volume, pH, creatinine (Cr), and Cr‐corrected bone resorption markers preflight, during spaceflight, and post‐spaceflight are shown in Table 2. Urine volume decreased rapidly during spaceflight, gradually increased post‐spaceflight, and returned to the baseline preflight value 1 year after return (R1yr) in both ARED+ALN and ARED groups. During spaceflight, pH gradually increased, became significantly higher than the preflight levels at day 180 of spaceflight (FD180) in the ARED group, and returned to the baseline level at R0. There was no significant difference in pH between the two groups. Daily excretion of urinary Cr remained almost constant throughout spaceflight in both groups with the exception of FD15 and FD120 when urinary Cr excretion was significantly greater than that at preflight in the ARED group. Change in daily urinary excretion of bone resorption markers, NTX and HP, from baseline is illustrated in Fig. 1. In the ARED group, urinary NTX and HP were significantly higher than baseline from FD15 to R0. In the ARED+ALN group, urinary NTX and HP were significantly lower than baseline on FD180 and R0. There were significant differences between two groups from FD15 to R30.

**Table 2:** Daily Urine Volume, pH, and Creatinine Excretion and Bone Resorption Markers Before, During, and After Spaceflight

Parameter	PRE	FD15	FD30	FD60	FD120	FD180	R0	R30	R1yr
Urine volume (L/d)									
ARED+ALN	2.3 ± 1.4	1.4 ± 0.8^a^	1.2 ± 0.6^a^	1.6 ± 0.8^a^	1.7 ± 0.8^a^	1.5 ± 1.0^a^	1.7 ± 0.7	1.9 ± 1.0	2.3 ± 1.0
ARED	2.4 ± 0.8	1.6 ± 0.5^b^	1.7 ± 0.4^b^	1.8 ± 0.5	2.2 ± 1.0	1.9 ± 0.7^b^	2.4 ± 0.8	2.1 ± 0.5	2.3 ± 0.7
pH									
ARED+ALN	6.0 ± 0.2	6.1 ± 0.4	6.1 ± 0.2	6.1 ± 0.4	6.1 ± 0.2	6.1 ± 0.3	5.8 ± 0.3	5.9 ± 0.5	6.1 ± 0.3
ARED	6.0 ± 0.2	6.0 ± 0.3	6.3 ± 0.4	6.1 ± 0.2	6.3 ± 0.4	6.3 ± 0.4^b^	5.9 ± 0.5	6.1 ± 0.3	6.3 ± 0.4
U‐Cr (mg/d)									
ARED+ALN	1810.8 ± 366.6	1960.3 ± 713.5	1618.9 ± 620.1	1987.3 ± 663.7	1915.8 ± 528.0	1893.4 ± 494.3	1890.1 ± 381.2	1754.1 ± 404.2	1788.6 ± 438.1
ARED	2063.2 ± 260.3	2387.5 ± 514.3^b^	2342.3 ± 587.6	2298.8 ± 902.1	2461.8 ± 954.4^b^	2324.6 ± 713.2	2044.4 ± 370.8	1950.8 ± 315.4	1887.2 ± 450.8
U‐Cr (mmol/d)									
ARED+ALN	16.0 ± 3.2	17.3 ± 6.3	14.3 ± 5.5	17.6 ± 5.9	16.9 ± 4.7	16.7 ± 4.4	16.7 ± 3.4	15.5 ± 3.6	15.8 ± 3.9
ARED	18.2 ± 2.3	21.1 ± 4.5^b^	20.7 ± 5.2	20.3 ± 8.0	21.8 ± 8.4^b^	20.5 ± 6.3	18.1 ± 3.3	17.2 ± 2.8	16.7 ± 4.0
U‐NTX (nmol/mmol Cr)									
ARED+ALN	23.3 ± 6.3	18.5 ± 4.9*	17.7 ± 4.7*	17.0 ± 6.4*	17.0 ± 5.7*	14.0 ± 6.8^a^, *	14.3 ± 9.5^a^, *	16.4 ± 5.8*	15.6 ± 5.7
ARED	25.8 ± 9.5	39.5 ± 10.5^b^	38.9 ± 14.6^b^	45.3 ± 14.7^b^	39.7 ± 18.9^b^	42.0 ± 11.3^b^	39.9 ± 16.3^b^	27.2 ± 9.8	26.0 ± 11.1
U‐HP (μg/mmol Cr)									
ARED+ALN	34.0 ± 13.0	26.7 ± 12.7*	40.8 ± 22.2*	27.0 ± 13.9*	25.9 ± 9.7*	21.1 ± 13.1^a^, *	20.4 ± 16.8^a^, *	26.8 ± 8.8	25.1 ± 11.6
ARED	50.4 ± 17.2	73.0 ± 15.8^b^	74.7 ± 25.8^b^	84.5 ± 28.2^b^	83.9 ± 34.7^b^	78.2 ± 20.3^b^	82.4 ± 22.4^b^	53.9 ± 14.6	36.7 ± 7.8^b^

Values are mean ± SD.

ARED = exercise; ARED+ALN = exercise plus alendronate; PRE = preflight; U‐Cr = urinary creatinine; U‐HP = urinary helical peptide; U‐NTX = urinary cross‐linked N‐telopeptide of type I collagen; SD = standard deviation.

^a^ *p* < 0.05 versus PRE in ARED+ALN.

^b^ *p* < 0.05 versus PRE in ARED.

* *p* < 0.05 between ARED+ALN and ARED. Contrast tests were conducted on the raw scale by a linear mixed effects model.

**Fig. 2:**
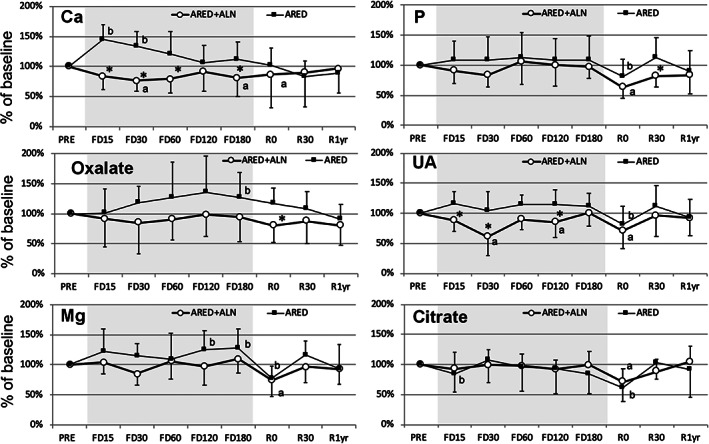
Percent changes from preflight in urine biochemistry. Dots indicate mean and error bars standard deviation of change (%) from preflight calculated using estimates from a linear mixed effects model. ^a^ *p* < 0.05 versus PRE in ARED+ALN, ^b^ *p* < 0.05 versus PRE in ARED. *p < 0.05 between the raw values of ARED and ARED+ALN. ARED = exercise alone; ARED+ALN = exercise plus alendronate; Ca = calcium; Mg = magnesium; P = phosphorus; UA = uric acid.

The temporal changes in urinary excretion of urolithiasis‐related factors from before spaceflight to 1 year after return were demonstrated in Table 3 and Fig. 2. In the ARED group, daily urinary calcium rapidly increased after the initiation of spaceflight with significant elevation at FD15 to FD30, but was not significantly different from preflight for the rest of the spaceflight. In the ARED+ALN group, urinary calcium was significantly lower than the baseline value at FD30, FD180, and R0. Urinary calcium was significantly different between the ARED and the ARED+ALN groups at FD15 to FD60 and FD180. There was no significant change from baseline in urinary phosphorus (P) excretion during spaceflight, but urinary P was significantly reduced in both groups relative to preflight at R0. There was significant difference between the two groups on R30. Urinary oxalate excretion showed a significant elevation from baseline at FD180 in the ARED group, with significant difference between the two groups on R0. Urinary UA during spaceflight was significantly reduced from baseline at FD30 and FD120 in the ARED+ALN group, and significant differences in urinary UA between the two groups were detected on FD15, FD30, and FD120. In both groups, urinary UA was significantly reduced at R0 relative to preflight, but returned to the preflight levels by R30 and R1yr.

**Table 3:** Temporal Changes in Urine Biochemistry

	PRE	FD15	FD30	FD60	FD120	FD180	R0	R30	R1yr
Ca (mg/d)									
ARED+ALN	268.1 ± 97.9	209.8 ± 92.7*	152.6 ± 41.1^a^, *	210.3 ± 93.9*	225.0 ± 97.2	202.8 ± 79.6^a^, *	200.6 ± 93.7^a^	211.9 ± 93.8	244.4 ± 111.8
ARED	224.1 ± 55.3	319.2 ± 91.5^b^	303.7 ± 93.7^b^	276.7 ± 121.2	243.0 ± 103.6	248.5 ± 97.0	226.5 ± 85.8	191.7 ± 86.3	197.1 ± 56.6
P (mg/d)									
ARED+ALN	1195.1 ± 413.7	1076.7 ± 417.1	960.5 ± 212.3	1219.9 ± 452.9	1179.7 ± 356.7	1141.3 ± 445.0	722.5 ± 193.2^a^	946.7 ± 215.7*	953.3 ± 320.5
ARED	1178.5 ± 194.2	1244.5 ± 276.5	1221.5 ± 297.1	1289.1 ± 468.3	1240.2 ± 350.3	1234.6 ± 499.8	917.9 ± 275.2^b^	1311.5 ± 307.4	1021.1 ± 300.8
Mg (mg/d)									
ARED+ALN	130.6 ± 60.9	131.2 ± 58.1	98.6 ± 25.8	128.5 ± 47.1	130.5 ± 49.3	142.1 ± 82.0	88.2 ± 23.2^a^	122.1 ± 48.0	117.4 ± 52.3
ARED	116.6 ± 37.0	134.7 ± 38.8	147.8 ± 27.9	130.6 ± 61.9	160.2 ± 35.4^b^	145.9 ± 53.1^b^	91.6 ± 41.8^b^	130.8 ± 33.2	117.1 ± 52.2
UA (mg/d)									
ARED+ALN	756.2 ± 256.0	644.5 ± 179.5*	388.6 ± 108.2^a^, *	678.3 ± 273.6	606.4 ± 156.9^a^, *	702.6 ± 299.4	510.7 ± 215.5^a^	674.9 ± 161.8	644.4 ± 127.3
ARED	716.7 ± 130.3	868.5 ± 221.5	762.5 ± 196.1	850.7 ± 264.2	845.3 ± 251.5	823.9 ± 274.9	610.8 ± 101.9^b^	837.4 ± 234.8	718.7 ± 233.2
Oxalate (mg/d)									
ARED+ALN	45.3 ± 13.6	38.5 ± 15.9	34.3 ± 24.1	39.2 ± 15.0	42.1 ± 10.3	41.8 ± 22.1	34.0 ± 8.2*	37.2 ± 14.6	33.9 ± 10.7
ARED	41.1 ± 15.9	39.6 ± 12.3	51.0 ± 25.0	52.6 ± 28.0	53.4 ± 14.8	55.3 ± 27.6^b^	47.7 ± 21.7	42.1 ± 9.8	36.2 ± 8.8
Citrate (mg/d)									
ARED+ALN	757.5 ± 227.9	724.8 ± 419.8	659.4 ± 115.0	766.4 ± 376.9	729.8 ± 280.9	794.7 ± 409.6	548.1 ± 265.5^a^	777.6 ± 293.0	787.0 ± 303.1
ARED	844.5 ± 342.9	680.2 ± 246.6^b^	876.0 ± 169.3	743.9 ± 303.3	782.1 ± 256.2	660.3 ± 222.7	475.0 ± 265.6^b^	711.9 ± 138.1	683.9 ± 284.2

Values are mean ± SD. Each value is indicated as daily excretion (mg/d).

ARED = exercise; ARED+ALN = exercise plus alendronate; Ca = calcium; Mg = magnesium; P = phosphorus; PRE = preflight; SD = standard deviation; UA = uric acid.

^a^ *p* < 0.05 versus PRE in ARED+ALN.

^b^ *p* < 0.05 versus PRE in ARED.

* *p* < 0.05 between ARED+ALN and ARED. Contrast tests were conducted on the raw scale by a linear mixed effects model.

As protective factors against urolithiasis, urinary magnesium (Mg) and citrate were measured. Urinary Mg in the ARED group was significantly elevated at FD120 and FD 180, whereas that in the ARED+ALN group remained stable during spaceflight. After spaceflight, urinary Mg of both groups was significantly decreased relative to preflight levels at R0, but returned to original levels at R30 and R1yr. Urinary citrate was similarly stable and was not significantly different between the two groups throughout the study. There was the exception of a significant reduction in urinary citrate at FD15 in the ARED group and at R0 in both groups compared to baseline (Fig. 2).

**Fig. 3:**
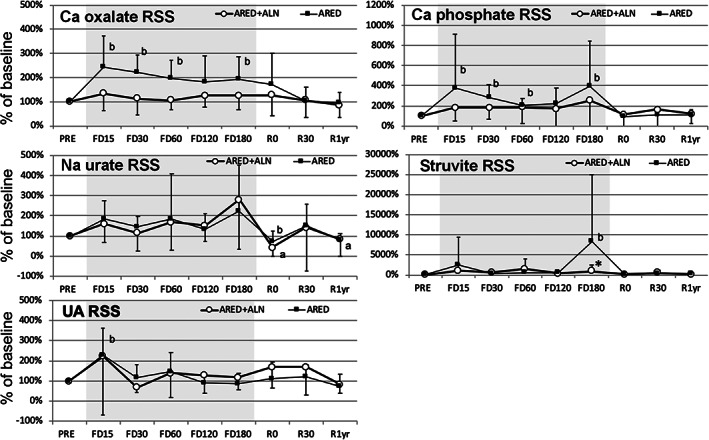
Percent changes from preflight in relative supersaturations. Dots indicate mean and error bars standard deviation of change (%) from preflight calculated using estimates from a linear mixed effects model. ^a^ *p* < 0.05 versus PRE in ARED+ALN, ^b^ *p* < 0.05 versus PRE in ARED. *p < 0.05 between the raw values of ARED and ARED+ALN. ARED = exercise alone; ARED+ALN = exercise plus alendronate; Na urate = monosodium urate; RSS = relative supersaturation; UA = uric acid.

To analyze the risk of renal stone formation, relative supersaturation (RSS) was calculated for each stone component (Fig. 3). RSS for calcium oxalate increased during spaceflight in the ARED group at FD15, FD30, FD60, and FD180, whereas it showed no significant change throughout the study in the ARED+ALN group. RSS for calcium phosphate was significantly increased from preflight value during spaceflight in the ARED group at FD15, FD30, FD60, and FD180, although the difference was not significant between the two groups. RSS for monosodium urate showed similar change in the two groups during spaceflight, and was significantly decreased from preflight levels at R0 in both groups. RSS for struvite was stable during spaceflight in both groups until FD120, but increased significantly in the ARED group at FD180, with significant difference between the two groups. RSS for UA was stable throughout the study, except for a significant increase from baseline in the ARED group at FD15 (Table 4).

**Table 4:** Temporal Changes in Values of Relative Supersaturation of Renal Stone Forming Salts

Parameter	PRE	FD15	FD30	FD60	FD120	FD180	R0	R30	R1yr
Calcium oxalate RSS									
ARED+ALN	2.1 ± 1.0	2.5 ± 1.1	2.3 ± 1.3*	2.1 ± 0.9*	2.2 ± 0.5	2.4 ± 1.2	2.4 ± 1.7	1.8 ± 0.8	1.7 ± 1.1
ARED	1.5 ± 0.9	2.9 ± 0.8^b^	3.0 ± 1.4^b^	2.6 ± 1.1^b^	2.3 ± 1.2	2.4 ± 0.9^b^	2.2 ± 1.7	1.3 ± 0.7	1.3 ± 0.5
Calcium phosphate RSS									
ARED+ALN	1.9 ± 1.1	2.6 ± 1.3	3.0 ± 2.1	2.4 ± 1.3	2.2 ± 1.2	2.7 ± 1.0	0.9 ± 0.7	1.3 ± 0.8	1.9 ± 1.9
ARED	1.3 ± 1.0	3.3 ± 2.4^b^	3.2 ± 1.2^b^	2.4 ± 1.6^b^	2.3 ± 1.0	2.9 ± 1.9^b^	0.9 ± 0.6	1.3 ± 1.0	1.4 ± 0.8
Sodium urate RSS									
ARED+ALN	3.9 ± 2.9	4.4 ± 3.0	3.6 ± 2.8	3.9 ± 2.6	4.1 ± 3.2	4.7 ± 2.5	1.0 ± 0.8^a^	3.1 ± 2.7	2.3 ± 2.1^a^
ARED	2.5 ± 2.7	3.6 ± 2.6	2.8 ± 1.4	3.1 ± 2.7	2.5 ± 1.4	3.0 ± 2.5^b^	1.1 ± 0.5^b^	3.0 ± 2.5	2.4 ± 3.2
Struvite RSS									
ARED+ALN	1.0 ± 0.9	3.1 ± 3.1	4.5 ± 5.5	3.0 ± 2.8	2.0 ± 2.1	2.7 ± 2.0*	0.3 ± 0.3	1.5 ± 1.6	1.1 ± 1.1
ARED	1.0 ± 1.6	2.4 ± 4.0	3.4 ± 3.6	1.4 ± 1.1	5.5 ± 9.1	9.0 ± 18.2^b^	0.5 ± 1.0	1.4 ± 1.7	2.0 ± 2.7
Uric acid RSS									
ARED+ALN	1.7 ± 0.9	2.3 ± 2.0	1.3 ± 0.9	2.0 ± 1.4	1.6 ± 0.8	1.9 ± 1.2	2.6 ± 1.9	2.2 ± 1.7	1.5 ± 1.5
ARED	1.4 ± 1.0	2.8 ± 1.5^b^	1.4 ± 0.9	1.9 ± 1.4	1.1 ± 0.7	1.3 ± 1.1	1.4 ± 1.0	1.6 ± 0.9	1.0 ± 0.8

Values are mean ± SD.

ARED = exercise; ARED+ALN = exercise plus alendronate; PRE = preflight; RSS = relative supersaturation; SD = standard deviation.

^a^ *p* < 0.05 versus PRE in ARED+ALN.

^b^ *p* < 0.05 versus PRE in ARED.

* *p* < 0.05 between ARED+ALN and ARED. Contrast tests were conducted on the raw scale by a linear mixed effects model.

**Fig. 4:**
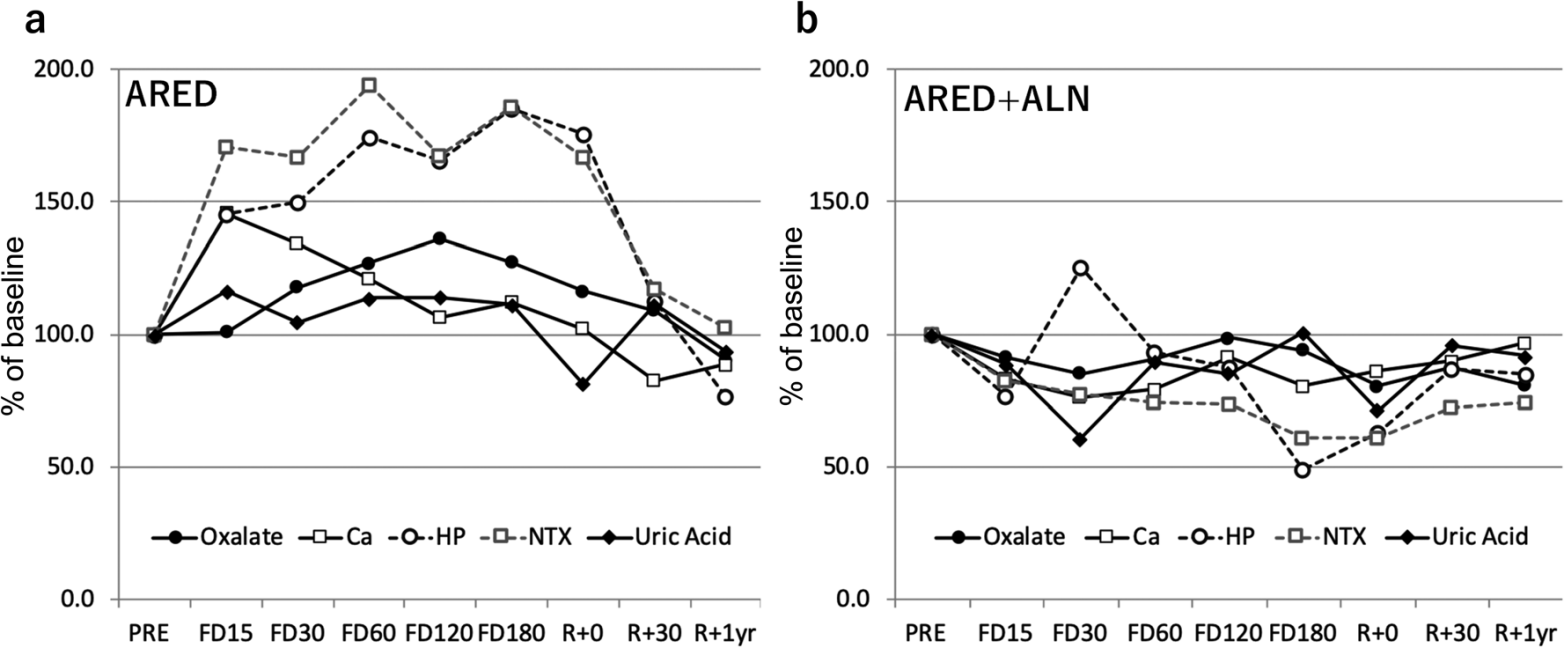
Percent changes in renal stone‐related factors and bone resorption markers plotted together in ARED group (*A*) and ARED+ALN group (*B*). Dots indicate mean of change (%) from preflight calculated using estimates from a linear mixed effects model. Ca = calcium; HP = helical peptide; Mg = magnesium; NTX = cross‐linked N‐telopeptide of type I collagen; P = phosphorus; UA = uric acid.

Overall, urinary excretion of calcium, oxalate, and UA, along with urinary bone resorption markers were greater in the ARED group relative to the ARED+ALN group, although statistically significant difference was observed for not all the time points during spaceflight (Figs. 1 and 2). Figure 4 displays all these markers over time. In the ARED group (Fig. 4*A*), urinary NTX and HP showed rapid increases and remained stable during spaceflight. Urinary calcium increased rapidly during the early period until FD15 and decreased gradually thereafter. Urinary oxalate and UA increased later during spaceflight and returned to preflight levels after return. In contrast, most of the markers decreased or remained stable throughout the study in the ARED + ALN group (Fig. 4*B*).

## Discussion

During spaceflight, there are clear benefits to the musculoskeletal system from resistive exercise,^(^ ^2^ ^)^ and from dietary countermeasures such as hydration and citrate supplementation in preventing risk of urinary calculi.^(^ ^3^, ^8^ ^)^ However, the present observations revealed that, even with ARED exercise, astronauts in the ISS demonstrated enhanced bone resorption and increased urinary calcium excretion during spaceflight. This is in agreement with the previous study that adequate nutrition and resistive exercise using ARED enhanced bone formation especially toward the later period of 6‐month spaceflight and alleviated the reduction in BMD, but could not prevent the enhancement of bone resorption with increased urinary calcium excretion.^(^ ^3^ ^)^ In the present study, administration of an anti‐resorptive, ALN, was able to reduce: (i) much of the observed spaceflight induced bone resorption, and (ii) some of the urinary calcium excretion throughout the spaceflight, as reported.^(^ ^13^, ^14^ ^)^ The reduction in bone resorption by ALN in ARED+ALN group was associated with lower urinary oxalate and UA excretion compared to those in ARED group, without change in citrate excretion. As a result, RSS for both calcium oxalate and calcium phosphate increased in ARED group during spaceflight, whereas there was no change in these parameters in ARED+ALN group. These results indicate that the enhanced bone resorption cannot be suppressed by resistive exercise alone using ARED, that the inhibition of bone resorption by ALN almost completely suppressed the increase in bone resorption markers and reduced urinary calcium excretion, and that the suppression of bone resorption by ALN also reduced the risk of calcium oxalate or calcium phosphate stones as shown by RSS. Thus, it is suggested that inhibition of bone resorption during spaceflight is effective not only in protecting bone, but also in driving down the risk of urolithiasis by reducing some of the urinary solutes which enhance renal stone formation.

Because the excretion of oxalate, UA and calcium was higher in the ARED group with enhanced bone resorption and was lower in the ARED+ALN group with suppressed bone resorption, there appears to be a relationship between urinary bone resorption markers and the excretion of calcium, oxalate, and UA. These results are consistent with the notion that enhanced bone resorption increases not only urinary calcium excretion, but also urinary oxalate and UA excretion. Having multiple urinary solutes elevated concurrently can also increase the risk of heterologous nucleation.

Bone is composed of matrix proteins and hydroxyapatite crystals deposited onto those matrix proteins. Upon bone resorption, hydroxyapatite is dissolved under the acid environment in osteoclastic resorption lacunae, and calcium, phosphate, and hydroxyl ions are released from bone. Because calcium is most abundant among them, the increased bone resorption is associated with increased release of calcium from bone with resultant increase in urinary calcium excretion. Bone matrix proteins consist of mostly type I collagen along with other matrix proteins including osteocalcin, osteonectin, matrix Gla protein, proteoglycans, etc. By the actions of cathepsin K released from osteoclasts, type I collagen is degraded into small peptides, and NTX and HP are among those peptides cleaved from type I collagen by cathepsin K. HP originates from helical portion of α1 chain of type I collagen at amino acids 620 to 633, ^620^Ala‐Hyp‐Gly‐Asp‐Arg‐Gly‐Glu‐Hyp‐Gly‐Pro‐Hyp‐Pro‐Ala^633^, which contains three hydroxyproline molecules.^(^ ^17^ ^)^ Because hydroxyproline can be metabolized to oxalate,^(^ ^18^ ^)^ the increase in type I collagen degradation products by the enhanced bone resorption may be the mechanism whereby urinary excretion of oxalate was increased. In fact, correlation between osteoporosis and urinary hydroxyproline excretion has been reported.^(^ ^19^ ^)^ Likewise, because type I collagen contains many glycine and aspartate molecules that can be metabolized to purine nucleotides, and because purine nucleotides are further metabolized to UA, increased excretion of these amino acids by the enhanced bone resorption can increase UA excretion. Thus, the increase in bone resorption may increase urinary excretion of not only calcium but also oxalate and UA. Because high urinary UA excretion is a risk factor for calcium oxalate stone formation,^(^ ^20^ ^)^ it is plausible to assume that the increase in urinary excretion of UA along with calcium and oxalate due to increased bone resorption increases the risk of urolithiasis in astronauts.

If the increase in urinary excretion of oxalate and UA along with calcium is due to the enhanced bone resorption in the astronauts, urinary excretion of oxalate, UA, and calcium should be decreased along with the reduction in bone resorption markers by ALN. In fact, as shown in Fig. 4, ALN in addition to ARED exercise reduced the excretion of these factors. These results are consistent with the notion that the suppression of bone resorption by ALN can reduce the risk of not only osteoporosis but also urolithiasis by reducing the excretion of risk factors for urolithiasis.

There are several limitations in the present study. First, the number of participants was small, and they were not randomly assigned to the groups. This has always been a limitation of studies on astronauts with the small study power. Therefore, we included the baseline in the model, to best represent the characteristics of participants. Nevertheless, due to the lack of statistical power, differences that might be clinically relevant and important might not have been detected. Likewise, because of the study design, the effect of ALN alone could not be assessed; therefore, it was not possible to determine if adding ALN to exercise was an additive or synergetic effect. However, it should be noted that exercise using iRED or ARED was, and continues to be, a required routine of all the astronauts under the weightless condition in the space. Nevertheless, the findings of this study can help generate hypotheses to test in further analytic studies. Second, we could not control the assignment of astronauts study participants to treatment groups; ie, each astronaut was given the option of ingesting ALN as a supplement to required exercise performance. Therefore, we were unable to randomize study participants based upon their age, sex, body mass index (BMI), or similar attributes. It should also be noted that data associated with astronaut age, sex, BMI, or with mission descriptors could potentially identify the astronaut participant and, therefore are reported as group mean values only. Third, clinical renal stone formation was evaluated only by ultrasonography. Recently in clinical diagnosis for renal stones, low‐dose noncontrast computed tomography (NCCT) has been recommended.^(^ ^21^ ^)^ The identification of renal stones by low‐dose NCCT in sensitivity and specificity to regular‐dose NCCT, and the radiation exposure of low‐dose NCCT (0.97–1.9 mSv) is about 70% lower than regular dose NCCT (4.5–5 mSv) and about twice that of abdominal plain radiography (0.5–1 mSv).^(^ ^22^ ^)^ Based on these data, because low‐dose NCCT is powerful in detecting microcalculi, we might be able to detect a difference in preventing renal calculi formation by ALN administration compared to ARED exercise alone by employing this imaging tool. There have been at least 14 urinary calculi events in astronauts,^(^ ^23^ ^)^ so the risk of stone formation is real, but this study used renal stone risk markers as surrogates, and did not detect the clinical event of a de novo renal stone in any astronaut study subject. This may speak well for both countermeasures evaluated, resistive exercise and ALN, in regard to reduction of clinical risk of calculus formation. Finally, we did not assess bone resorptive inflammatory cytokines, such as interleukin 1β (IL‐1β), IL‐6, and tumor necrosis factor α (TNF‐α). We have reported that these cytokines can trigger inflammatory macrophage activation and induce renal stone formation in mice and humans.^(^ ^24^, ^25^, ^26^, ^27^, ^28^, ^29^ ^)^ In addition, IL‐1β and TNF‐α are increased during spaceflight, with a trend for increased IL‐6.^(^ ^30^ ^)^ Further studies are needed to assess changes in these blood and urinary cytokines as risk factors for renal stone formation during spaceflight.

In conclusion, the present results demonstrate that astronauts on long‐term spaceflights are at risk of the formation of urinary calcium oxalate and calcium phosphate stones through an increase in urinary excretion of oxalate and UA from degradation product of type I collagen as well as calcium due to enhanced bone resorption, and that, by inhibiting bone resorption using anti‐resorptive agents, not only the reduction in bone mass, but also the increase in urinary calculus risk, are attenuated in astronauts. Our findings suggest that enhanced bone resorption can be another risk factor for renal stone formation during spaceflight, and by adding anti‐resorptive agents, eg, ALN, to existing countermeasures such as hydration and citrate supplementation, the risk of urinary calculus formation can be further reduced.

## Conflict of Interest

AO, TM, HO, TI, TK, TY, KK, AL, JS, ES, JJ, and LS have no conflict of interest related to this study.

## Data Availability

The data supporting the results in this paper cannot be shared by readers because of the NASA and JAXA data protection policy, as well as protecting privacy of participated astronauts.
